# Integrated Multi-omics Analysis of Hub Genes and miRNA Interactions in Hypertrophic Cardiomyopathy

**DOI:** 10.2174/0113892029363785250311084956

**Published:** 2025-03-26

**Authors:** Huanhuan Hu, Ziheng Yu, Kongjie Lu, Hui Hu, Lang Deng

**Affiliations:** 1 Huzhou Central Hospital, Fifth School of Clinical Medicine of Zhejiang Chinese Medical University, Huzhou, 313000, China;; 2 Huzhou Central Hospital, Affiliated Central Hospital of Huzhou University, Huzhou, 313000, China

**Keywords:** HCM, hub genes, diagnosis, miRNAs, treatment, pathways

## Abstract

**Background:**

Hypertrophic Cardiomyopathy (HCM) is a complex cardiac disorder marked by the thickening of the heart muscle.

**Methods:**

HCM and normal control cell lines were cultured in DMEM with 12.5% FBS and 1% penicillin-streptomycin at 37°C and 5% CO_2_. Differentially expressed genes (DEGs) were identified from GSE32453, GSE53408, and GSE113439 datasets using the limma package in R. Hub genes were determined through protein-protein interaction (PPI) network and Cytoscape analysis and validated *via* Reverse Transcription Quantitative Polymerase Chain Reaction (RT-qPCR) and Western blot analysis. Gene enrichment, miRNA predictions, drug prediction, and molecular docking analyses were conducted for functional enrichment and to explore hub gene-associated drugs.

**Results:**

To identify DEGs and hub genes implicated in HCM, we analyzed three Gene Expression Omnibus (GEO) datasets (GSE32453, GSE53404, and GSE1134439), extracting the top 1000 DEGs. Venn analysis revealed 21 common down-regulated genes. PPI analysis identified these six as key hub genes, including Iron Response Element Binding Protein 2 (IREB2), Protein Tyrosine Phosphatase, Non-Receptor Type 11 (PTPN11), IQ Motif Containing GTPase Activating Protein 1 (IQGAP1), Phosphoglucomutase 2 (PGM2), DIS3 RNA Exonuclease 3' to 5' (DIS3), Glutamine-Fructose-6-Phosphate Transaminase 1 (GFPT1) in HCM patients. Gene enrichment analysis highlighted the involvement of these genes in cellular functions such as energy metabolism and growth factor signaling, suggesting their role in the disease's progression. Validation using an additional dataset (GSE36961) confirmed significant down-regulation of all hub genes in HCM samples, supported by Receiver Operating Characteristic (ROC) curve analysis that demonstrated their diagnostic potential. Furthermore, miRNA analysis identified six up-regulated miRNAs (miR-124, miR-29b, miR-330, miR-34a, miR-375, and miR-451) that likely contribute to the dysregulation of these hub genes. Drug prediction analysis identified various potential therapeutic compounds targeting these hub genes. Molecular docking revealed favorable binding affinities, supporting the therapeutic potential of these drugs in modulating hub gene activity.

**Discussion:**

These findings demonstrate that HCM progression involves coordinated downregulation of hub genes and miRNA-mediated dysregulation of metabolic and signaling pathways. The integration of bioinformatics, validation assays, and drug docking suggests a strong translational potential for biomarker discovery and targeted therapy.

**Conclusion:**

Our findings suggest that IREB2, PTPN11, IQGAP1, PGM2, DIS3, and GFPT1 hub genes and their associated regulatory pathways may serve as biomarkers and therapeutic targets for HCM, potentially improving diagnosis and treatment strategies.

## 1 INTRODUCTION

Hypertrophic Cardiomyopathy (HCM) is a genetically driven cardiac disorder characterized by the abnormal thickening of the heart muscle, predominantly affecting the left ventricle [1, 2]. This condition often leads to impaired diastolic filling, increased risk of arrhythmias, and, in severe cases, sudden cardiac death [3, 4]. As a leading cause of sudden cardiac death in young athletes, HCM represents a significant challenge for both clinicians and researchers [5-7]. Despite extensive research efforts, the pathophysiology of HCM remains complex and not fully elucidated. The identification of differentially expressed genes (DEGs) and hub genes associated with HCM is critical for understanding the underlying mechanisms of the disease and identifying potential therapeutic targets.

HCM is often caused by mutations in genes encoding sarcomeric proteins, which are integral to the contractile machinery of the heart [8-10]. Key genes implicated in HCM include β-Myosin Heavy Chain (MYH7), Cardiac Troponin T (TNNT2), and Cardiac Troponin I (TNNI3) [8, 11, 12]. Mutations in these genes lead to defects in the sarcomere, resulting in altered contractility and hypertrophy [8, 11, 12]. However, recent studies have expanded our understanding beyond these canonical sarcomeric genes, highlighting the role of non-sarcomeric genes and regulatory networks in the disease. Additionally, as highlighted in genomic research, the absence of standardized datasets and the difficulties in establishing sequencing pipelines have limited the wider adoption of genomics [13]. Therefore, combining genomic analysis with transcriptomic analysis can provide a more comprehensive understanding of the molecular basis of the disease.

Several studies have investigated DEGs in HCM, uncovering a range of genes involved in various biological processes such as fibrosis, inflammation, and metabolism. For instance, Transforming Growth Factor Beta 1 (TGF-β1): Known for its role in fibrosis, TGF-β1 has been shown to be upregulated in HCM, contributing to myocardial fibrosis and hypertrophy [14]. Fibronectin 1 (FN1): An extracellular matrix protein that plays a role in fibrosis, FN1 has been reported to be elevated in HCM, reflecting increased fibrotic activity [15]. Collagen Type I Alpha 1 Chain (COL1A1): Increased expression of COL1A1 in HCM tissues suggests enhanced collagen deposition, which is a hallmark of fibrosis [16]. Myosin Light Chain 2 (MYL2): Elevated levels of MYL2 have been observed in HCM, implicating alterations in contractile function [17]. Atrial Natriuretic Peptide (ANP): Upregulation of ANP in HCM is indicative of atrial stretch and stress, contributing to the pathophysiology of the disease [18]. B-Type Natriuretic Peptide (BNP): Similar to ANP, BNP levels are increased in HCM and are used as a marker of heart failure and disease severity [19]. While these studies have provided valuable insights into the molecular changes associated with HCM, they also have limitations. Many of these studies involve small sample sizes, which may not capture the full spectrum of gene expression changes in HCM. Furthermore, the findings are often not replicated across different patient cohorts or validated through *in vitro* experiments [20], limiting the generalizability of the results.

Recent advances in high-throughput technologies, such as microarray and RNA sequencing, have facilitated more comprehensive analyses of gene expression profiles in HCM [7, 21, 22]. By leveraging these technologies, researchers can identify a broader set of DEGs and construct gene interaction networks that provide a more holistic view of the disease. For example, studies utilizing RNA sequencing (RNA-Seq) [23-25] have identified novel DEGs and regulatory pathways that were not previously recognized, offering new insights into the molecular mechanisms underlying HCM [21, 26] and other diseases like chronic inflammatory pathologies [27].

In this study, we hypothesized that HCM is driven not only by mutations in canonical sarcomeric genes, such as MYH7, TNNT2, and TNNI3 but also by alterations in the expression of non-sarcomeric genes and regulatory networks. These non-sarcomeric factors, in conjunction with sarcomeric gene mutations, may contribute to the complex pathophysiology of the disease, including processes like fibrosis, inflammation, and altered cellular signaling.

In this study, we aim to answer the scientific question: What are the key molecular players, including non-sarcomeric genes, involved in the pathogenesis of HCM, and how can they be targeted for therapeutic intervention? By integrating multi-omics data, network analyses, and molecular experiments, we seek to provide a more comprehensive understanding of the molecular basis of HCM, which will ultimately guide the development of novel therapeutic approaches.

## 2 MATERIALS AND METHODS

### 2.1 Cell Culture

A total of four HCM cell lines, including AC16, iPSC-derived cardiomyocytes, HEK293, and HCM, along with three normal control cell lines derived from healthy human heart tissues, including HCFs, HCMECs, and NHCF, were obtained from Merck-Millipore, USA. These cells were cultured in DMEM supplemented with 12.5% FBS and 1% penicillin-streptomycin under incubation conditions of 37°C and 5% CO_2_.

### 2.2 Acquisition of Gene Expression Profiles

The expression profile data of mRNA for the datasets GSE32453, GSE53408, and GSE113439 were obtained from the Gene Expression Omnibus (GEO, http://www.ncbi.nlm.nih.gov/geo/) database [28]. GSE32453, GSE53408, and GSE113439 datasets provide detailed gene expression profiles across HCM, which is essential for identifying DEGs and potential biomarkers for the disease. Moreover, these datasets include samples relevant to the study’s focus on disease mechanisms, with GSE32453 containing expression data for 8 patients and 5 control samples, GSE53408 for 12 patients and 11 controls, and GSE113439 for 15 patients tissue samples and 11 controls. The expression profiles were carefully pre-processed to ensure data consistency across different platforms. The details of these datasets, including sample characteristics, are summarized in Table **1**.

### 2.3 DEGs Identification

The limma package in R software was utilized to identify DEGs between normal and HCM samples. The criteria for DEG selection were set as |log2FC| ≥ 1, a *P*-value < 0.05, and a false discovery rate (FDR) < 0.05 to reduce the likelihood of false positives. A Venn diagram package was then applied to pinpoint the common DEGs across datasets. Additionally, the “heatmap” package in R was used to generate heatmaps for visualizing gene expression patterns.

### 2.4 Protein-protein Interaction (PPI) Network Analysis of DEGs and Hub Genes Identification

To predict the physical and functional interactions between proteins encoded by hub genes, PPI networks were constructed using the STRING (Search Tool for the Retrieval of Interacting Genes/Proteins) online database [29, 30]. A combined score >0.4 was applied as the cutoff criterion to ensure the reliability of the predicted interactions. The PPI networks were then visualized and further analyzed using Cytoscape software (Version 3.10.2), where the CytoHubba plugin was employed to calculate the degree of connectivity for each protein node [31]. The degree metric, which reflects the number of direct interactions a protein has with other proteins in the network, was used to rank the importance of each node. In this study, the top six nodes with the highest degree values were identified and designated as hub genes.

### 2.5 Gene Enrichment Analysis

Changes in the expression of intracellular pathways may play a significant role in the pathogenesis of the disease [32]. In the current work, both Gene Ontology (GO) and KEGG (Kyoto Encyclopedia of Genes and Genomes) analyses were performed utilizing the ClusterProfiler package in R. An adjusted *p*-value cutoff of < 0.05 was applied to maintain statistical rigor and ensure that only significantly enriched terms and pathways were considered for interpretation.

### 2.6 Validation of Hub Genes Using Additional HCM Dataset

To validate the expression of the identified hub genes, we used the GSE36961 dataset from the GEO database, which contains gene expression profiles from 106 patients and 19 normal control samples (Table **2**).

### 2.7 Prediction and Validation of Hub Genes-associated miRNAs

miRNet is a comprehensive platform designed for network-based analysis and functional interpretation of microRNAs (miRNAs), [33] genes, and other molecular entities [34]. It integrates data from multiple public databases, providing insights into miRNA-gene interactions, disease associations, and pathway involvement. In this study, we utilized the miRNet database to predict miRNAs associated with the identified hub genes. The database enabled us to identify miRNA-gene interactions based on a combination of experimentally validated and predicted data. Each interaction was assigned a prediction score, reflecting the confidence level of the association. From the list of predicted miRNAs, the top-ranking miRNA, based on its prediction score, was selected for further analysis.

Next, total RNA was extracted from four HCM cell lines (AC16, iPSC-derived cardiomyocytes, HEK293, and HCM) and three normal control cell lines (HCFs, HCMECs, and NHCF) using the miRNeasy Mini Kit (Qiagen, Cat. No. 217004), following the manufacturer’s instructions. RNA concentration and purity were assessed using a NanoDrop spectrophotometer.

For reverse transcription, cDNA synthesis was performed using the TaqMan™ Advanced miRNA cDNA Synthesis Kit (Thermo Fisher Scientific, Cat. No. A28007). The reaction was set up according to the protocol, which includes poly(A) tailing, adapter ligation, reverse transcription, and amplification of miRNAs. Reverse Transcription Quantitative Polymerase Chain Reaction (RT-qPCR) was performed using TaqMan™ Fast Advanced Master Mix (Thermo Fisher Scientific, Cat. No. 4444557) and specific TaqMan™ miRNA Assays for miR-124 (Cat. No. 4427975), miR-29b (Cat. No. 4427975), miR-330 (Cat. No. 4427975), miR-34a (Cat. No. 4427975), miR-375 (Cat. No. 4427975), and miR-451 (Cat. No. 4427975). Reactions were performed in 96-well plates on the QuantStudio™ 5 Real-Time PCR System (Thermo Fisher Scientific, Cat. No. A28134).

The relative expression of each miRNA was calculated using the comparative Ct method (ΔΔCt), normalizing to U6 small nuclear RNA as an internal control. Each sample was run in triplicate, and data were analyzed using QuantStudio software.

### 2.8 RT-qPCR Analysis of Hub Genes on HCM Cell Lines

RT-qPCR was performed with the TaqMan™ Fast Advanced Master Mix (Thermo Fisher Scientific, Cat. No. 4444557) and specific TaqMan™ Gene Expression Assays for the following hub genes: IREB2 (Cat. No. 4331182, Assay ID: Hs00241623_m1), PTPN11 (Cat. No. 4331182, Assay ID: Hs00936927_m1), IQGAP1 (Cat. No. 4331182, Assay ID: Hs00958226_m1), PGM2 (Cat. No. 4331182, Assay ID: Hs00157803_m1), DIS3 (Cat. No. 4331182, Assay ID: Hs00232735_m1), and GFPT1 (Cat. No. 4331182, Assay ID: Hs00366471_m1). Reactions were conducted in triplicate on the QuantStudio™ 5 Real-Time PCR System (Thermo Fisher Scientific, Cat. No. A28134). Relative expression levels of the hub genes were calculated using the comparative Ct method (ΔΔCt), with normalization to the housekeeping gene GAPDH (Cat. No. 4331182, Assay ID: Hs02758991_g1), and data analysis was performed using QuantStudio software.

The relative expressions of the proteins were calculated by normalizing their band intensity to that of the housekeeping protein (GAPDH) using the following formula:

Relative Expression = Target Protein Band Intensity (Corrected) / GAPDH Band Intensity (Corrected)

### 2.9 Western Blot Analysis

Initially, cells from four HCM cell lines (AC16, iPSC-derived cardiomyocytes, HEK293, HCM) and three normal control cell lines (HCFs, HCMECs, NHCF) were harvested, rinsed with cold PBS (Catalog number: 10010049), and lysed using RIPA buffer (Thermo Fisher Scientific, Catalog number: 89901) supplemented with protease inhibitors (Thermo Fisher Scientific, Halt™ Protease and Phosphatase Inhibitor Cocktail, Catalog number: 78440). The lysates were placed on ice for 30 minutes, with occasional vortexing. The samples were centrifuged at 12,000 rpm for 10 minutes at 4°C, and the supernatant containing the proteins was collected. Protein concentration was quantified using the Pierce™ BCA Protein Assay Kit (Catalog number: 23225).

Equal amounts of protein (20-30 µg) were mixed with LDS Sample Buffer (Thermo Fisher Scientific, Catalog number: B0007) and heated at 95°C for 5 minutes. The samples were then loaded onto a NuPAGE™ 4-20% Bis-Tris Gel (Catalog number: NP0323BOX) and run at 120V using NuPAGE™ MOPS SDS Running Buffer (Catalog number: NP0001) until the proteins were sufficiently separated. Proteins were transferred onto a PVDF membrane (Immobilon^®^-P PVDF, Catalog number: IPVH00010) using the Trans-Blot Turbo Transfer System (Bio-Rad, Catalog number: 1704150) at 100V for 1 hour.

The membrane was blocked with the blocking reagent provided in the WesternBreeze™ Chemiluminescent Immunodetection Kit (Thermo Fisher Scientific, Catalog numbers: WB7106 for anti-rabbit and WB7104 for anti-mouse) for 1 hour at room temperature on a shaker. After blocking, the membrane was incubated overnight at 4°C with primary antibodies specific to IREB2 (Catalog Number: PA1-16544), PTPN11 (Catalog Number: TA890142), IQGAP1 (Catalog Number: PA5-95625), PGM2 (Catalog Number: PA5-118160), DIS3 (Catalog Number: PA5-50123), GFPT1 (Catalog Number: PA5-76473), GAPDH (Catalog Number: MA5-15738), diluted in blocking buffer (typically 1:500 to 1:2000, depending on the antibody).

The next day, the membrane was washed three times with the provided washing buffer from the WesternBreeze™ kit. The HRP-conjugated secondary antibody (Goat Anti-Rabbit IgG (H+L) HRP-conjugated Secondary Antibody, Catalog Number: 31460) was applied to the membrane for 1 hour at room temperature. Following this, the membrane was washed three more times using the washing buffer from the kit, followed by a 5-minute incubation with the chemiluminescent substrate from the same WesternBreeze™ kit. Protein bands were detected using the Amersham Hyperfilm™ ECL (Catalog number: 28906836) or a chemiluminescent imaging system such as the iBright™ Imaging System (Thermo Fisher Scientific, Catalog number: A44114). Band intensities were quantified using ImageJ software to compare the expression of the hub gene proteins across the HCM and control cell lines.

### 2.10 Hub Genes-associated Expression Regulatory Drugs

The DSigDB (Disease Signature Database) is a comprehensive resource that catalogs gene expression signatures associated with various diseases. It integrates data from numerous studies to provide insights into disease mechanisms, biomarkers, and therapeutic targets. Researchers use DSigDB to explore gene expression profiles, identify disease-specific signatures, and facilitate the development of novel diagnostic and therapeutic strategies. In this work, the DSigDB database was searched to explore hub genes-associated expression regulatory drugs.

### 2.11 Molecular Docking

First, we employed the PubChem database to obtain the PDB structures of targeted drugs [35]. Then, we employed the ENSEMBLE database [36] to obtain FASTA sequences of the proteins encoded by hub genes. After that, we employed the SwissModel database [37] to model the PDB structures of the proteins using FASTA sequences. In the end, we used the SeamDock tool [38] to conduct molecular docking, thereby forecasting the binding sites and binding strength between proteins and drugs.

### 2.12 Statistics

DEGs were identified using the limma package in R with criteria |log2FC| ≥ 1 and *P* < 0.05. Common DEGs across datasets were determined using Venn diagrams, and heatmaps were generated with the “heatmap” package in R. PPI networks were analyzed with STRING (score >0.4) and visualized in Cytoscape, where hub genes were ranked by connectivity degree. Receiver Operating Characteristics (ROC) curve analysis was conducted to analyze the diagnostic potential. GO and KEGG analyses were performed using the ClusterProfiler package in R with an adjusted *P*-value threshold of < 0.05. RT-qPCR data were analyzed using the comparative Ct method (ΔΔCt) [39], with normalization to U6 and GAPDH. Statistical significance was assessed using Student’s t-test. *P*-values < 0.05, 0.01, and 0.001 were considered significant.

## 3 RESULTS

### 3.1 Identification of DEGs and Hub Genes Among HCM Patients as Compared to Controls

**Fig. (1):**
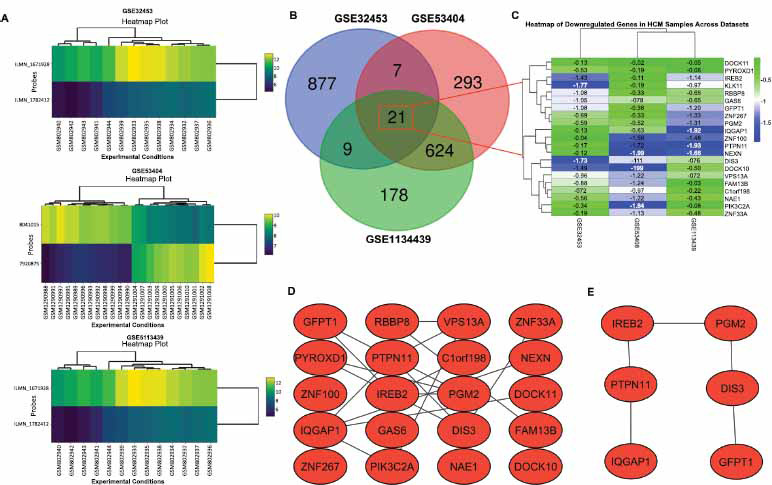
Exploring differentially expressed genes (DEGs) and hub genes hypertrophic cardiomyopathy (HCM) patients using integrated approach. (**A**) Expression heatmap plots of HCM samples across three Gene Expression Omnibus (GEO) datasets (GSE32453, GSE53404, and GSE1134439). (**B**) Venn diagram showing the overlap of DEGs across the three datasets. (**C**) Heatmap showing the expression of 21 down-regulated genes in HCM samples across all three datasets. (**D**) Protein-protein interaction (PPI) network of the 21 down-regulated genes. (**E**) Subnetwork of key down-regulated hub genes from the PPI analysis.

The identification of DEGs and hub genes in HCM patients compared to controls was done by combining 3 (GSE32453, GSE53404, and GSE1134439) GEO datasets (Fig. **1A**). The top 1000 DEGs from each dataset were obtained and subjected to Venn analysis. The selection of the top 1000 DEGs helps prioritize genes that are likely to have a substantial impact on disease pathogenesis, making them more likely to serve as potential biomarkers or therapeutic targets. Venn analysis results showed that the majority of DEGs were dataset-specific, while a smaller subset of DEGs is shared across datasets. Notably, 21 down-regulated genes were common among all three datasets (Fig. **1B** and **C**), suggesting these may be core DEGs strongly associated with HCM pathogenesis. Consequently, a PPI network analysis of DEGs was conducted with the STRING database. A total of 21 nodes and 36 edges were screened in the PPI network (Fig. **1D**). Nodes with high topological scores were considered as hub genes, including Iron Response Element Binding Protein 2 (IREB2), Protein Tyrosine Phosphatase, Non-Receptor Type 11 (PTPN11), IQ Motif Containing GTPase Activating Protein 1 (IQGAP1), Phosphoglucomutase 2 (PGM2), DIS3 RNA Exonuclease 3' to 5' (DIS3), Glutamine-Fructose-6-Phosphate Transaminase 1 (GFPT1) playing important roles in HCM (Fig. **1E**).

### 3.2 Gene Enrichment Analysis of Hub Genes

**Fig. (2):**
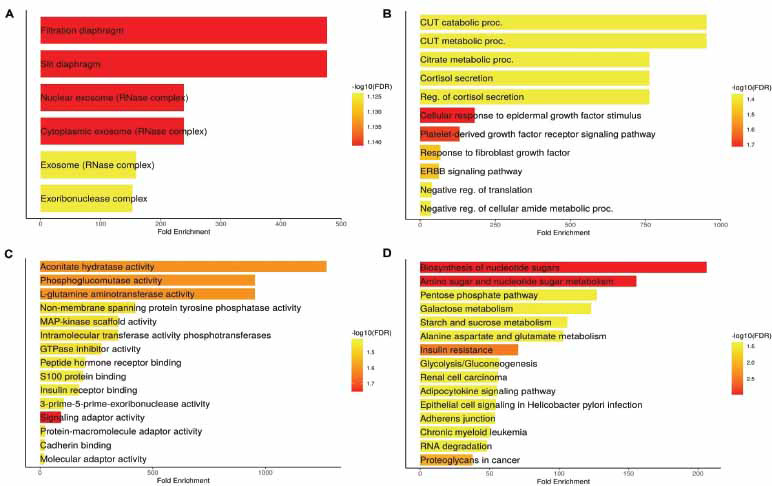
Functional enrichment analysis of hub genes in hypertrophic cardiomyopathy (HCM). (**A**) Cellular component (CC) enrichment analysis of hub genes. (**B**) Biological process (BP) enrichment analysis. (**C**) Molecular function (MF) enrichment analysis. (**D**) KEGG pathway enrichment analysis. *P*-value < 0.05.

Gene enrichment analysis of hub genes was conducted using the ClusterProfiler package in R. The analysis covers multiple aspects of cellular function, including cellular components (CC), biological processes (BP), molecular functions (MF), and metabolic pathways, offering a comprehensive view of the potential roles these hub genes may play in the pathophysiology of HCM. In Fig. (**2A**), the enrichment analysis of CC reveals significant involvement of several structural and functional units within the cell. Among the most enriched are the filtration diaphragm and slit diaphragm, which are essential components in kidney podocytes, suggesting a potential link between cardiac and renal function in HCM patients. Additionally, components of the nuclear and cytoplasmic exosome complexes, which are involved in RNA degradation and processing, are also enriched (Fig. **2A**). This implies that the regulation of RNA metabolism could be a key factor in the development or progression of HCM, as dysregulated RNA processing is often associated with cellular stress and disease. Fig. (**2B**) presents the enriched MF. The analysis identifies significant involvement in metabolic processes, such as citrate metabolism, and pathways related to cortisol secretion (Fig. **2B**). These findings suggest that altered metabolic states, including disrupted energy production and stress hormone regulation, could play a role in HCM pathology. Furthermore, several growth factor signaling pathways, such as those related to epidermal growth factor and platelet-derived growth factor, are also enriched (Fig. **2B**), indicating that abnormal signaling and cellular growth responses might contribute to cardiac hypertrophy and fibrosis, both hallmark features of HCM.

In Fig. (**2C**), the focus is on enriched BP, revealing activities related to energy metabolism, such as aconitate hydratase activity and phosphoglucomutase activity, which are key components of the TCA cycle and carbohydrate metabolism (Fig. **2C**). These findings further suggest that metabolic dysfunction, particularly in pathways related to energy production and glucose metabolism, may be central to HCM development. Additionally, functions related to protein signaling and binding, including MAP-kinase scaffold activity and insulin receptor binding, are enriched (Fig. **2C**), highlighting potential disruptions in cellular signaling mechanisms that regulate growth, metabolism, and stress responses. Finally, Fig. (**2D**) highlights the enriched pathways associated with the hub genes, many of which are linked to metabolic processes. These include pathways involved in the biosynthesis and metabolism of nucleotide sugars, pentose phosphate pathways, and starch and sucrose metabolism (Fig. **2D**). These pathways are critical for maintaining cellular energy and structural components, and their enrichment suggests that alterations in these metabolic processes may contribute to the energy deficits and structural abnormalities seen in HCM. The enrichment of insulin resistance and pathways related to cell adhesion, such as adherens junctions (Fig. **2D**), also suggests that metabolic dysfunction and altered cell-cell interactions may be important factors in the disease’s progression.

### 3.3 Validation of Hub Genes Using Additional HCM Dataset

**Fig. (3):**
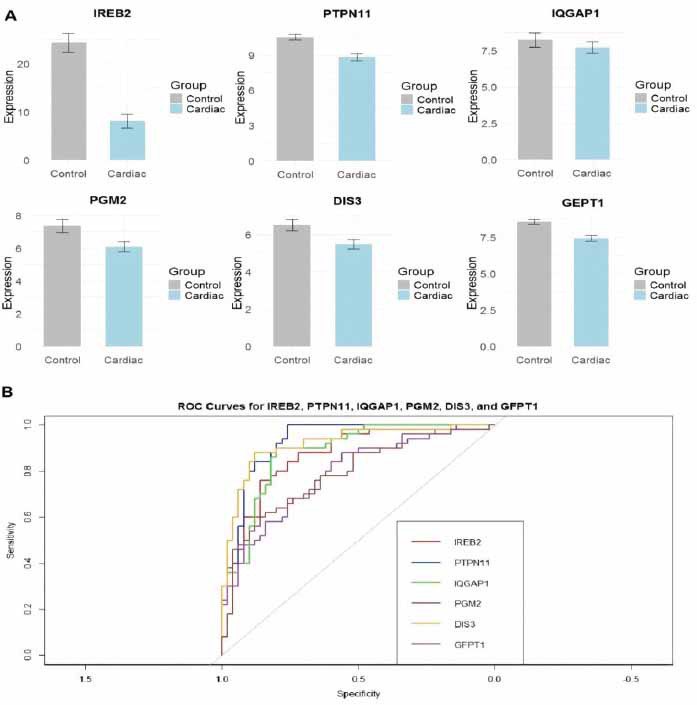
Validation of hub genes in hypertrophic cardiomyopathy (HCM) samples using additional Gene Expression Omnibus (GEO) dataset (GSE36961) and receiver operating characteristic (ROC) curve analysis. (**A**) Bar plots showing the expression levels of six hub genes (IREB2, PTPN11, IQGAP1, PGM2, DIS3, and GFPT1) in control and cardiac tissue samples. (**B**) ROC curve analysis for the six hub genes (IREB2, PTPN11, IQGAP1, PGM2, and DIS3, GFPT1). The different colored lines represent individual genes, and the area under the curve (AUC) values indicate their diagnostic potential. *P*-value < 0.05.

We further validated the expression of the hub genes using an additional HCM dataset (GSE36961). Fig. (**3A**) shows the expression levels of IREB2, PTPN11, IQGAP1, PGM2, DIS3, and GFPT1 in both HCM and control groups. All hub genes, including PTPN11, IQGAP1, PGM2, DIS3, and GFPT1, exhibited a significant (*p* < 0.05) reduction in expression in HCM samples compared to the controls (Fig. **3A**). Fig. (**3B**) shows the ROC curves for the hub genes, providing a measure of their sensitivity and specificity in distinguishing between HCM and control samples. The area under the curve (AUC) values indicates how well each gene can differentiate between the two groups. IREB2 shows the highest AUC, indicating strong potential as a diagnostic biomarker for HCM (Fig. **3B**). Other genes such as PTPN11, IQGAP1, PGM2, DIS3, and GFPT1 also demonstrate good predictive power, though with varying levels of diagnostic utility based on their ROC curves (Fig. **3B**).

### 3.4 Prediction and Validation of Hub Genes-associated miRNAs

**Fig. (4):**
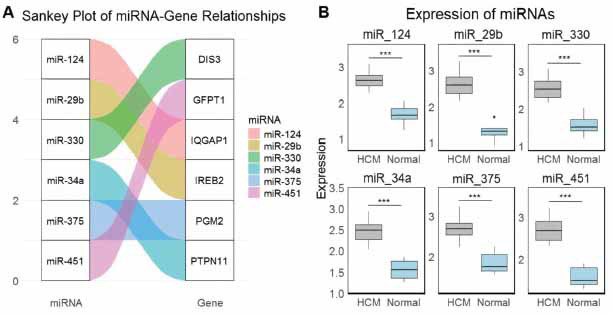
miRNA-gene relationships and expression levels of miRNAs in hypertrophic cardiomyopathy (HCM). (**A**) Sankey plot of miRNA-gene relationships sourced from miRNet database: A Sankey diagram representing the interactions between miRNAs and their target genes. (**B**) Expression of miRNAs in HCM and normal control cell lines: Box plots showing the expression levels of miR-124, miR-29b, miR-330, miR-34a, miR-375, and miR-451 in HCM and normal control samples. ***p < 0.001.

Firstly, hub genes-associated miRNAs [23] were predicted using the miRNet database. In Fig. (**4A**), the Sankey plot illustrates the relationships between hub genes and their predicted miRNAs. The miRNAs, including miR-124, miR-29b, miR-330, miR-34a, miR-375, and miR-451, were shown to regulate the expression of hub genes (IREB2, PTPN11, IQGAP1, PGM2, DIS3, and GFPT1) highlighting potential regulatory interactions in HCM. In Fig. (**4B**), the expression levels of the miRNAs were validated using RT-qPCR in HCM (n = 04) *versus* normal control (n = 03) cell lines. The results demonstrate significantly higher expression of miR-124, miR-29b, miR-330, miR-34a, miR-375, and miR-451 in HCM cells compared to normal controls (*p* < 0.001 for all miRNAs) (Fig. **4B**). This suggests that these miRNAs were up-regulated in HCM and may contribute to the dysregulation of their associated hub genes, potentially playing a crucial role in the pathogenesis of HCM.

### 3.5 Expression Validation of Hub Genes on HCM Cell Lines *via* RT-qPCR and Western Blot

**Fig. (5):**
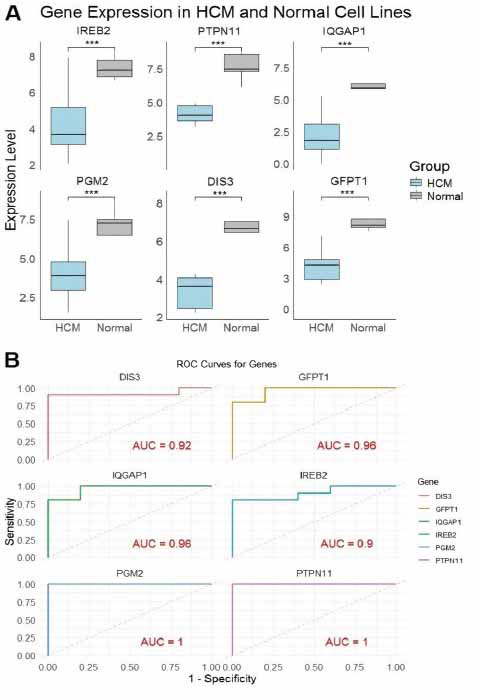
Expression validation, diagnostic performance, and potential drug interactions of hub genes in hypertrophic cardiomyopathy (HCM). (**A**) Gene expression in HCM and normal control cell lines: Box plots showing the expression levels of six genes (IREB2, PTPN11, IQGAP1, PGM2, DIS3, and GFPT1) in HCM and normal control cell lines. (**B**) Receiver Operating Characteristic (ROC) curves for hub genes: ROC curves evaluating the diagnostic performance of the six genes in distinguishing HCM from normal samples. The area under the curve (AUC) values are indicated for each gene, with high diagnostic accuracy shown for PGM2 (AUC = 1), PTPN11 (AUC = 1), IQGAP1 (AUC = 0.96), GFPT1 (AUC = 0.96), DIS3 (AUC = 0.92), and IREB2 (AUC = 0.9). ***p < 0.001.

**Fig. (6):**
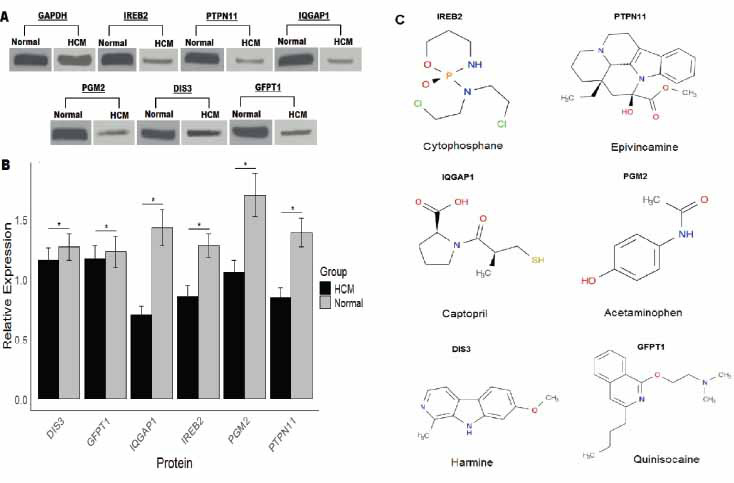
Western blot analysis of proteins encoded by hub genes in normal and hypertrophic cardiomyopathy (HCM) cells. (**A**) Western blot bands of GAPDH, IREB2, PTPN11, IQGAP1, PGM2, DIS3, and GFPT1 proteins in normal and HCM cells. (**B**) Bar graph representing the relative expression of each protein, normalized to GAPDH, in normal and HCM groups. (**C**) Potential drug interactions for hub genes: Chemical structures of compounds potentially interacting with the hub genes. Cytophosphane (IREB2), Epivincamine (PTPN11), Captopril (IQGAP1), Acetaminophen (PGM2), Harmine (DIS3), and Quinisocaine (GFPT1) are shown as potential therapeutic agents targeting these genes. *p < 0.05.

The expression of hub genes was validated in HCM (n = 04) and normal control (n = 03) cell lines using RT-qPCR and Western blot analyses. The results of RT-qPCR analysis demonstrate that all six hub genes (IREB2, PTPN11, IQGAP1, PGM2, DIS3, and GFPT1) were significantly down-regulated in HCM cells compared to normal controls (*p* < 0.001), validating their potential role in the pathogenesis of HCM (Fig. **5A**). Furthermore, Fig. (**5B**) presents ROC curves for these genes, with area under the curve (AUC) values ranging from 0.90 to 1.00. The high AUC values for DIS3 (0.92), GFPT1 (0.96), IQGAP1 (0.96), IREB2 (0.90), PGM2 (1.00), and PTPN11 (1.00) indicate strong predictive power in distinguishing HCM from normal samples, supporting their potential as biomarkers for HCM diagnosis (Fig. **5B**). Additional expression validation analyses *via* Western blot analysis also reveal that IQGAP1, PGM2, IREB2, and PTPN11 exhibit significantly lower protein expression in HCM cells than in Normal cells (Fig. **6A** and **B**).

### 3.6 Hub Genes-associated Expression Regulatory Drugs

Drug prediction analysis was performed using the DSigDB database to identify potential drugs targeting the hub genes (IREB2, PTPN11, IQGAP1, PGM2, DIS3, and GFPT1) to enhance their expressions. The predicted drugs include Cytophosphane for IREB2, Epivincamine for PTPN11, Captopril for IQGAP1, Acetaminophen for PGM2, Harmine for DIS3, and Quinisoquine for GFPT1 (Fig. **6C**). These findings suggest potential therapeutic targets for the treatment of HCM, based on the identified hub genes and their associated drugs. Next, we performed a molecular docking analysis of identified drugs with the proteins encoded by hub genes to validate their binding abilities. In Fig. (**7A**), Cytochophane exhibits a binding affinity score of -6.3 kcal/mol with IREB2, indicating a moderately favorable interaction where the drug binds within a key pocket of the protein. Fig. (**7B**) shows Epivincamine docking with PTPN11, yielding a stronger binding score of -7.0 kcal/mol, suggesting a robust interaction and potential activation of PTPN11 activity. In Fig. (**7C**), Captopril interacts with IQGAP1 with a binding score of -6.8 kcal/mol, highlighting a significant docking fit, which implies potential interference with cellular processes mediated by IQGAP1. Fig. (**7D**) presents Acetaminophen docking with PGM2, showing a lower binding score of -6.1 kcal/mol, indicating a more moderate interaction within the shallow pockets of the PGM2 protein. Fig. (**7E**) highlights Harmine, which exhibits the strongest interaction with DIS3, reflected by a binding score of -7.5 kcal/mol, suggesting a significant activation potential. Lastly, Fig. (**7F**) shows Quinisquine docking with GFPT1 with a binding score of -6.1 kcal/mol, representing a moderate interaction.

## 4 DISCUSSION

Hypertrophic cardiomyopathy (HCM) is a complex genetic disorder characterized by the thickening of the heart muscle, primarily affecting the left ventricle [40-42]. It is often associated with mutations in sarcomeric proteins that disrupt normal cardiac function, leading to symptoms such as chest pain, dyspnea, and an increased risk of sudden cardiac death [43]. As of 2023, the understanding of HCM pathogenesis has advanced, highlighting the involvement of both genetic and environmental factors in disease progression [44, 45]. Despite these advances, there is still much to learn about the molecular mechanisms underlying HCM and the identification of potential therapeutic targets.

Our study aimed to identify DEGs and hub genes in HCM patients by analyzing three independent GEO datasets (GSE32453, GSE53404, and GSE1134439). We identified 21 common down-regulated DEGs across all datasets, with key hub genes, including IREB2, PTPN11, IQGAP1, PGM2, DIS3, and GFPT1, through PPI network analysis. These genes are implicated in various cellular processes such as metabolic pathways, cell signaling, and protein interactions, suggesting their significant role in HCM pathogenesis.

Previous studies have also explored gene expression patterns in HCM; however, our research expands on these findings by integrating multiple datasets to identify common DEGs, which are more likely to be robust biomarkers. For instance, studies have reported alterations in genes involved in cardiac metabolism and cell signaling, which aligns with our identification of hub genes like PGM2 and IQGAP1, which play roles in glucose metabolism and intracellular signaling, respectively [46-48]. Furthermore, our gene enrichment analysis revealed that these hub genes are involved in critical biological processes such as cellular metabolism and RNA degradation, consistent with earlier findings that metabolic dysfunction contributes to HCM progression [49-51].

In particular, IREB2, a gene involved in iron homeostasis [44, 52, 53], was identified as a significant hub gene in our analysis. Disruptions in iron metabolism have been associated with cardiac diseases, including HCM, where iron overload or deficiency can exacerbate cardiac remodeling [54, 55]. The downregulation of IREB2 in HCM patients, as observed in our study, supports this connection and suggests that iron metabolism may play a more significant role in HCM than previously thought [56, 57]. This finding is consistent with recent research highlighting iron dysregulation as a contributing factor to cardiac hypertrophy [58].

Our study also identified PTPN11 as another critical hub gene. PTPN11, which encodes the protein SHP-2, is a tyrosine phosphatase involved in regulating various cellular signaling pathways, including growth factor signaling. Previous studies have linked PTPN11 mutations with cardiac dysfunctions, including hypertrophy and heart failure, reinforcing its relevance in HCM [59]. Interestingly, while PTPN11 mutations typically result in gain-of-function effects, our data suggest downregulation in HCM, highlighting the complexity of its role in cardiac pathology [60, 61]. This contrast may be due to differences in disease stages or compensatory mechanisms that emerge during HCM progression.

Furthermore, our molecular docking analysis predicted potential drugs that could target these hub genes, offering new avenues for therapeutic intervention. Harmine, which exhibited the strongest binding affinity with DIS3, has previously been studied for its role in modulating cellular growth and apoptosis, which are critical in HCM's pathological remodeling process [62-64]. Our findings suggest that Harmine could potentially modulate DIS3 activity and offer therapeutic benefits, though further experimental validation is necessary. Similarly, Cytophosphane, Epivincamine, and Captopril demonstrated favorable binding with IREB2, PTPN11, and IQGAP1, respectively, indicating their potential as candidates for pharmacological intervention in HCM. Notably, Captopril is already a well-known angiotensin-converting enzyme (ACE) inhibitor used to manage hypertensive heart conditions, including HCM, supporting our prediction [65-67].

Comparing our findings with those reported in other studies, we observed both consistencies and novel insights. While previous research has frequently emphasized sarcomeric protein mutations and alterations in calcium handling as primary drivers of HCM [44, 45, 68-71], our study highlights a broader range of molecular mechanisms, including metabolic dysregulation and RNA processing, suggesting that HCM may involve more widespread cellular dysfunction than previously understood. For instance, the enrichment of pathways related to nucleotide sugar metabolism and insulin resistance further underscores the importance of metabolic health in HCM pathophysiology [71-74]. This broader view aligns with recent studies suggesting that HCM is not solely a sarcomeric disease but also involves significant contributions from metabolic and signaling pathways [68, 75].

Concerning the novelty of the study, unlike previous reports that predominantly focused on sarcomeric protein mutations and calcium handling, our research highlights the involvement of metabolic dysfunction and RNA processing pathways, offering a broader understanding of HCM. Key hub genes such as PGM2, IQGAP1, and IREB2 were identified, suggesting that metabolic and iron homeostasis disruptions play critical roles in HCM progression. Additionally, we validated our findings using HCM cell lines through molecular experiments, further strengthening the relevance of these genes. Furthermore, our molecular docking analysis proposed potential therapeutic agents, providing a novel avenue for future HCM treatments.

This study has several strengths, including the integration of four independent GEO datasets (GSE32453, GSE53404, GSE1134439, and GSE36961), which enhances the reliability and generalizability of the findings. By identifying 21 common DEGs and key hub genes, including IREB2, PTPN11, IQGAP1, PGM2, DIS3, and GFPT1 hub genes, the study sheds light on critical cellular processes involved in HCM beyond the well-documented sarcomeric protein mutations. Furthermore, the inclusion of molecular docking analysis adds value by predicting potential therapeutic agents, including Harmine, Cytophosphane, Epivincamine, and Captopril, the latter of which is already used clinically for hypertensive heart conditions, reinforcing the practical relevance of these predictions. Another strength is the emphasis on metabolic pathways and RNA degradation, expanding the understanding of HCM beyond traditional views of it as a purely sarcomeric disease.

## STUDY LIMITATIONS

However, the study is limited by the lack of experimental validation. While the bioinformatics approach is robust, further experimental studies, such as *in vitro* or *in vivo* functional assays, are necessary to confirm the biological relevance of the identified hub genes and predicted drug interactions. Additionally, the cross-sectional nature of the datasets does not account for disease progression, which may influence gene expression profiles.

## CONCLUSION

In conclusion, our research significantly contributes to the expanding understanding of HCM as a multifaceted disorder with intricate molecular mechanisms. By integrating multiple independent datasets and performing robust bioinformatic analyses, we identified key hub genes—IREB2, PTPN11, IQGAP1, PGM2, DIS3, and GFPT1—implicated in essential cellular processes such as metabolic regulation, RNA degradation, and intracellular signaling. These findings broaden the current perspective on HCM pathogenesis, which has traditionally focused on sarcomeric mutations, by highlighting the critical roles of metabolic dysfunction and RNA processing. Additionally, our molecular docking predictions revealed potential therapeutic candidates such as Harmine, Cytophosphane, Epivincamine, and Captopril, offering new avenues for drug development. Captopril, an established ACE inhibitor, supports the relevance of targeting metabolic and signaling pathways for HCM management. These insights hold promise for the development of targeted treatments, particularly for patients who do not respond well to conventional therapies. Despite these promising findings, further experimental validation through *in vitro* and *in vivo* studies is essential to confirm the biological relevance of these hub genes and drug interactions. Nonetheless, our study lays a strong foundation for future research aimed at devising innovative therapeutic strategies to improve outcomes for HCM patients.

## Figures and Tables

**Fig. (7):**
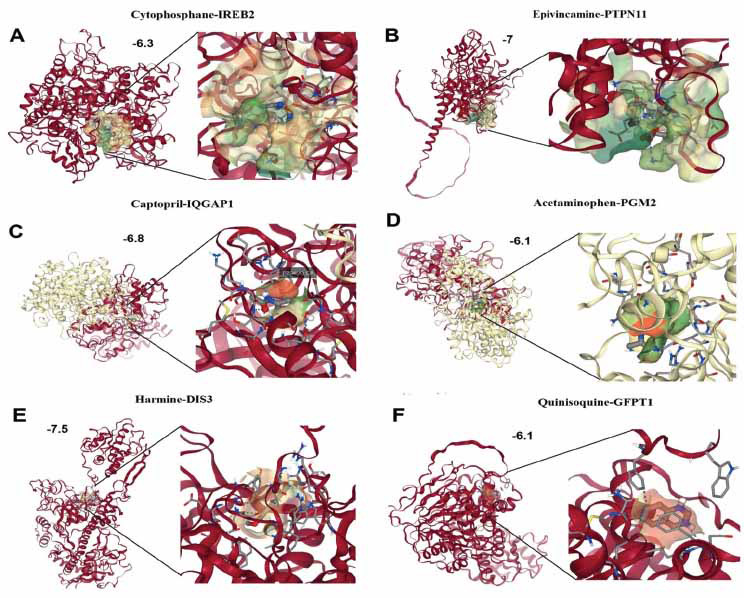
Molecular docking of potential therapeutic compounds with protein encoded by hub genes in hypertrophic cardiomyopathy (HCM). (**A**) Cytophosphane-IREB2 Interaction: Cytophosphane binds to the active site of IREB2 with a docking score of -6.3, showing key interactions with surrounding residues. (**B**) Epivincamine-PTPN11 Interaction: Epivincamine docks into the active site of PTPN11, with a binding affinity score of -7, suggesting strong interaction with the gene's active site. (**C**) Captopril-IQGAP1 Interaction: Captopril binds to IQGAP1 with a docking score of -6.8, highlighting its potential inhibitory effects through interactions with critical active site residues. (**D**) Acetaminophen-PGM2 Interaction: Acetaminophen shows moderate binding to PGM2, with a docking score of -6.1, indicating potential therapeutic relevance. (**E**) Harmine-DIS3 Interaction: Harmine binds strongly to DIS3 with a docking score of -7.5, interacting with multiple residues within the active site. (**F**) Quinisocaine-GFPT1 Interaction: Quinisocaine docks into GFPT1’s binding site with a docking score of -6.1, indicating potential inhibition through its interaction with the enzyme's active site.

**Table 1:** Summary of GSE32453, GSE53408, and GSE113439 datasets with diagnoses, sample sizes, and platforms.

**Dataset**	**Diagnosis**	**Number of Samples**	**Platform**
GSE32453	Controls	5	GPL570 (Microarray)
	Patients	8	
GSE53408	Controls	11	GPL10558 (Microarray)
	Patients	12	
GSE113439	Controls	11	GPL6244 (Microarray)
	Patients	15	

**Table 2:** Summary of the GSE36961 dataset with diagnoses, sample sizes, and platforms.

**Dataset**	**Diagnosis**	**Number of Samples**	**Platform**
GSE36961	Controls	19	GPL570 (Microarray)
	Patients	106	

## Data Availability

The datasets analyzed in this study, including GSE32453, GSE53408, GSE113439, and GSE36961, are publicly available in the Gene Expression Omnibus (GEO) database. These datasets can be accessed through the following link: https://www.ncbi.nlm.nih.gov/geo/. Data related to other molecular experiments can be provided by the corresponding author upon reasonable request.

## LIST OF ABBREVIATIONS

ACE: Angiotensin-converting Enzyme
ANP: Atrial Natriuretic Peptide
AUC: Area Under the Curve
BNP: B-Type Natriuretic Peptide
BP: Biological Processes
CC: Cellular Components
COL1A1: Collagen Type I Alpha 1 Chain
DEGs: Differentially Expressed Genes
DSigDB: Disease Signature Database
FDR: False Discovery Rate
FN1: Fibronectin 1
GEO: Gene Expression Omnibus
GFPT1: Glutamine-Fructose-6-Phosphate Transaminase 1
GO: Gene Ontology
HCM: Hypertrophic Cardiomyopathy
IQGAP1: IQ Motif Containing GTPase Activating Protein 1
IREB2: Iron Response Element Binding Protein 2
KEGG: Kyoto Encyclopedia of Genes and Genomes
MF: Molecular Functions
MYL2: Myosin Light Chain 2
PGM2: Phosphoglucomutase 2
PPI: Protein-protein Interaction
PTPN11: Protein Tyrosine Phosphatase, Non-Receptor Type 11
ROC: Receiver Operating Characteristic
RT-qPCR: Reverse Transcription Quantitative Polymerase Chain Reaction
TGF-β1: Transforming Growth Factor Beta 1
